# Interaction Study on Garlic and Atorvastatin with Reference to Nephrotoxicity in Dyslipidaemic Rats

**DOI:** 10.4103/0971-6580.72678

**Published:** 2010

**Authors:** G. Dilip Reddy, A. Gopala Reddy, G. Srinivasa Rao, C. Haritha, K. Jyothi

**Affiliations:** Department of Pharmacology and Toxicology, College of Veterinary Science, Hyderabad, India; 1 Department of Pharmacology and Toxicology, NTR College of Veterinary Science, Gannavaram, Krishna Dt., Andhra Pradesh - 521 102, India

**Keywords:** Atorvastatin, garlic, kidney

## Abstract

A total of 56 male *Sprague dawley* rats of uniform weight and age were randomly divided into seven groups consisting of eight rats in each group. Groups 1, 2, and 3 served as plain control, dyslipidaemic control (DL), and atorvastatin control, respectively. Groups 4, 5, 6, and 7 received 1, 0.5, 0.25, and 0.75% fresh garlic w/w in feed, respectively in addition to the high-fat and high-cholesterol diet and administered with atorvastatin orally for 12 weeks at the rate of 10, 5, 7.5, and 2.5 mg/kg b.wt., respectively. Plasma creatinine was estimated at 4-week intervals, whereas histopathology, electron microscopy, and estimation of TBARS concentration in kidney were conducted at the end of experiment. The TBARS concentration in DL was significantly (*P*<0.05) increased when compared with groups 1, 3, 6, and 7. On histopathological examination, kidney sections of group 3 had mild degenerative changes in the tubules with fatty change in few tubules, while groups 4 and 5 exhibited mild-to-moderate degenerative and fatty changes in tubules with inter tubular hemorrhages. The electron microscopy of group 2 showed hypertrophy of Bowman’s capsule, while that of group 4 showed secretary deposits in the cytoplasm. The interaction studies on kidney indicated that high dose of atorvastatin + garlic has negative safety profile when compared with groups having low dose of statin and high dose of garlic.

## INTRODUCTION

Dyslipidemias are major risk factors for atherosclerosis and its associated conditions such as coronary heart disease, ischemic cerebrovascular disease and peripheral vascular disease.[1] The drugs that are available to treat dyslipidemias include the statins (3-hydroxy-3-methylglutaryl coenzyme A reductase inhibitors), bile acid-binding resins, nicotinic acid (*niacin*), fibric acid derivatives, and the cholesterol absorption inhibitor *ezetimibe*.[1] The effectiveness of atorvastatin in normalizing dyslipidaemia has been proved in many studies.[2,3] The usage of herbal therapies along with prescription and over the counter medications is increasing day by day. Three of the ten most widely selling herbal medicines in the developed countries, namely preparations of *Allium sativum*, *Aloe barbadensis*, and *Panax* sp. are available in India.[4]

In both animal and human studies, garlic has been reported to lower cholesterol, triglycerides, and change blood lipoproteins, and to affect coagulation parameters.[5,6] Although popular belief about the herbal products is that many of these preparations are considered natural and safe, they require attention for potential risk as they are pharmacologically active. Most of these herbal remedies can interact with allopathic drugs, resulting in altered activity and toxicity. Herbs like garlic compete with other agents for metabolism by CYP450s and or inactivate P450 enzymes, affecting the bioavailability of certain coadministered drugs, leading to potentially severe clinical manifestations.[7] Keeping the above facts in view, an experimental study was planned to study the interaction of garlic and atorvastatin in different dose proportions in dyslipidaemic rats with respect to nephrotoxicity.

## MATERIALS AND METHODS

After an acclimatization period of 3 weeks, 56 male *Sprague dawley* rats of uniform age and weight were randomly divided into seven Groups of eight rats in each. Group 1 was kept as normal control and remaining six Groups were fed with diet containing 14% beef tallow and 1% cholesterol for six weeks to induce dyslipidaemia. After induction of dyslipidaemia, the experimental schedule was as follows: Group 1: Normal control; Group 2: Dyslipidaemic control (DL); Group 3: DL + Atorvastatin (10 mg/kg b.wt. orally) control; Group 4: DL + Atorvastatin (10 mg/kg b.wt. orally) + Garlic (1% in the feed w/w); Group 5: DL + Atorvastatin (5 mg/kg b.wt. orally) + Garlic (0.5% in the feed w/w); Group 6: DL + Atorvastatin (7.5 mg/kg b.wt. orally) + Garlic (0.25% in the feed w/w); and Group 7: DL + Atorvastatin (2.5 mg/kg b.wt. orally) + Garlic (0.75% in the feed w/w). Garlic treatment was initiated two weeks before the first oral dose of atorvastatin.

Blood samples were collected at monthly intervals and plasma was separated for estimation of creatinine by using diagnostic kits (Qualigens Pvt. Ltd., Mumbai), and kidney samples were collected at the end of experiment for estimation of thiobarbituric acid reacting substances (TBARS)[8] after homogenization. Pieces of kidney samples were collected in 10% formal saline for histological studies. After fixing in formalin, the tissues were processed according to the method described by Culling[9] and stained with H and E stain. Kidney samples were collected and fixed in 3% glutaraldehyde in 0.05 M phosphate buffer (pH 7.2) for 24 hours at 4°C and post-fixed with 2% aqueous osmium tetroxide in the same buffer for 1 hour for transmission electron microscopy. Subsequently, the samples were dehydrated in a series of graded alcohol and infiltrated and embedded in Araldite 6005 resin. Ultrathin sections (50 – 70 nm thickness) were cut with a glass knife on a Leica Ultra cut UCT-GA-D/E-1/00 ultra microtome and mounted on grids. The sections were further stained with saturated aqueous uranyl acetate and counter stained with 4% lead citrate[10] and observed at various magnifications under a transmission electron microscope (Model: Hitachi, H-7500).

## RESULTS AND DISCUSSION

**Figure 1:**
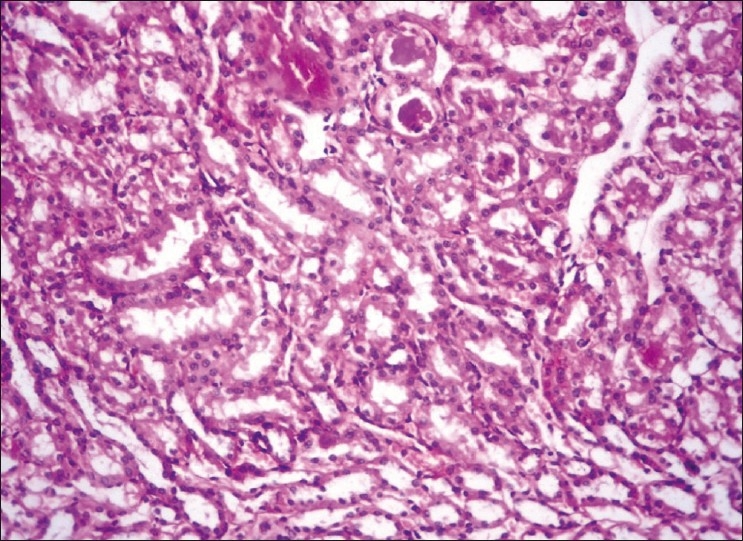
Kidney showing hyaline casts with moderate fatty change H and E, ×200 (Group-2)

**Figure 2:**
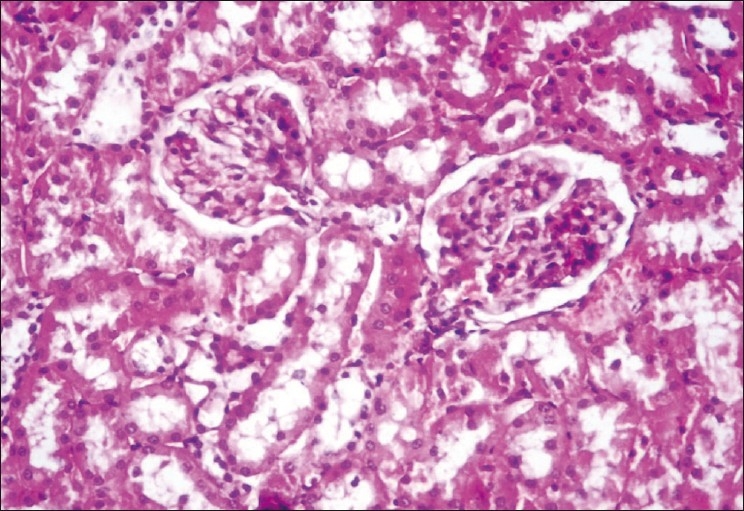
Kidney showing moderate fatty change and moderate degenerative changes in tubules with intertubular hemorrhages H and E, ×400 (Group-4)

**Figure 3:**
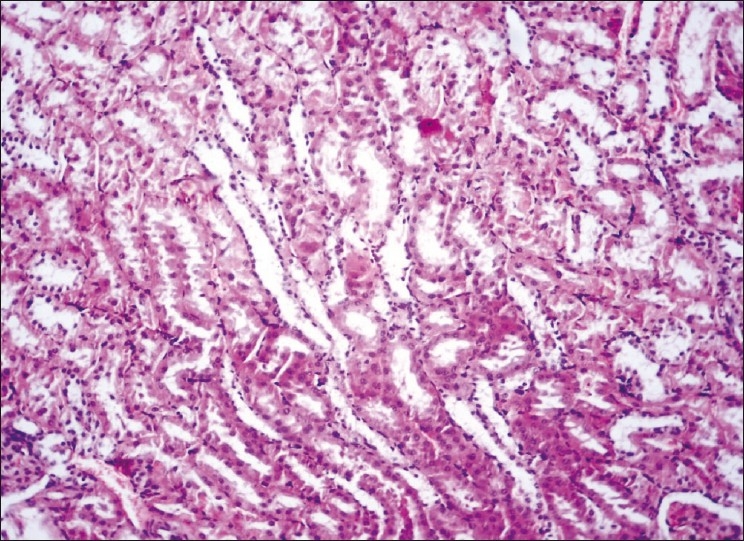
Kidney showing mild degenerative and fatty change in the tubules H and E, ×200 (Group-3)

**Figure 4:**
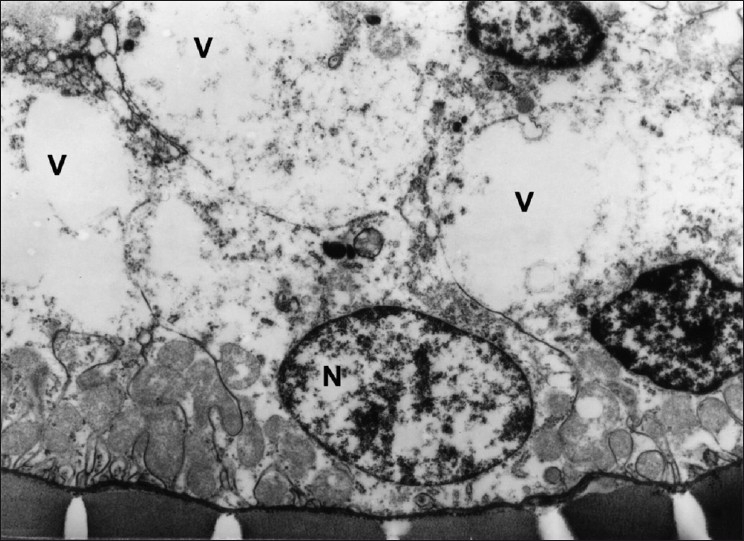
Transmission electron micrograph of DCT showing vacuolation (V) and ruptured cells. (Group 2) ×5490

**Figure 5:**
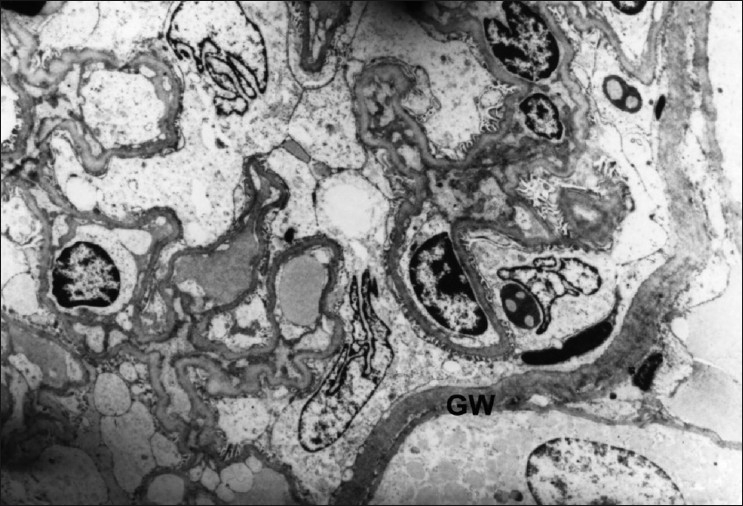
Transmission electron micrograph of glomerulus showing thickened endothelial and glomerular walls (GW). (Group-3) ×3294

**Figure 6:**
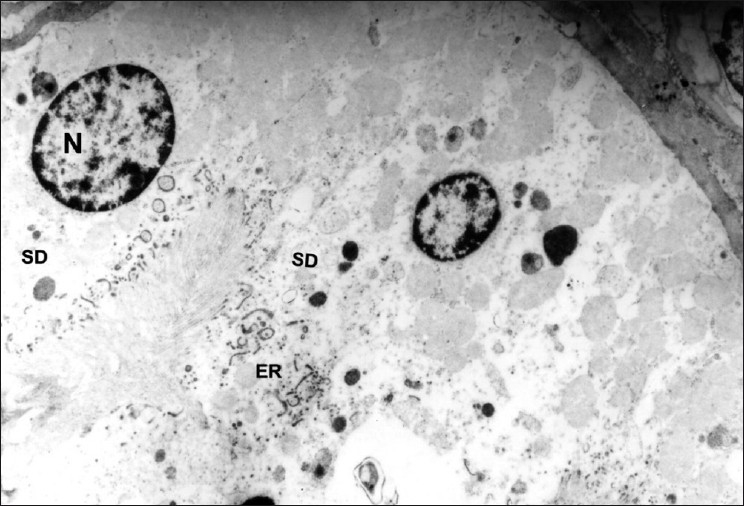
Transmission electron micrograph of PCT showing detached endoplasmic reticulum (ER) and secretory deposits (SD) in the cytoplasm. (Group-4) ×4575

The concentrations of plasma creatinine and TBARS in kidney were determined to assess the possibility of renal damage, if any, due to different treatments. The plasma creatinine concentration increases significantly when renal function is below 30% of its original ability.[11] The plasma creatinine concentration (mg/dl) of the normal control group was significantly (*P*<0.05) lower (ranged from 0.635 ± 0.026 to 0.651 ± 0.035) than those of DL (0.839 ± 0.018 to 0.849 ± 0.036) throughout the experiment [Table 1]. The treatment Groups 3 to 7 showed significant (*P*<0.05) increase at the end of fourth week, while the treatment Groups 4 and 5 showed significantly (*P*<0.05) higher concentrations of plasma creatinine at the end of eighth (0.791 ± 0.032 and 0.797 ± 0.052, respectively) and 12^th^ week (0.823 ± 0.037 and 0.807 ± 0.033, respectively) when compared with control (Group 1) and the plasma creatinine concentrations of Groups 3, 6, and 7 were comparable with that of control during same period. The statin control Group (3) showed a significant reduction in plasma creatinine concentration on eighth week (0.734 ± 0.027) when compared with its basal value (0.833 ± 0.039 mg/dl). Groups 4 to 6 displayed no significant change in the plasma creatinine concentrations throughout the study, whereas Group 7 exhibited significant decrease (*P*<0.05) from its base value (0.823 ± 0.029) at the end of 12^th^ week (0.656 ± 0.037). The creatinine concentration was found highest in DL (Group 2), showed hyaline casts with mild to moderate fatty change [Figure 1], followed by the Group 4 which exhibited moderate to severe fatty change, mild to moderate degenerative changes in tubules with inter tubular hemorrhages with normal glomeruli [Figure 2]. The creatinine concentration was lowest in Group 7. Kidney sections of Group 3 had mild degenerative changes in the tubules with fatty change in few tubules [Figure 3]. Similar changes were seen in Group 5, while Group 6 showed a milder degree of changes, and the sections of Group 7 exhibited a tendency towards normal morphology. The electron microscopy of kidney (Group 2) showed hypertrophy of Bowman’s capsule due to accumulation of fat. Afferent and efferent arteries thickened due to endothelial fat accumulation, while distal convoluted tubule (DCT) exhibited rupture of cells with apical vacuolation [Figure 4]. Glomerulus of Group 3 showed fat accumulation and thickened wall [Figure 5], while that of Group 4 showed secretary deposits, possibly of atorvastatin in the cytoplasm and detached endoplasmic reticulum [Figure 6].

**Table 1:** Creatinine concentration in plasma and TBARS concentration in kidney in different groups of rats

Groups	Plasma creatinine (mg/dl)		TBARS (nM/mg tissue)	
	4^th^ week	8^th^ week	12^th^ week	12^th^ week
Control	0.635 ± 0.026^aA^	0.641 ± 0.047^aA^	0.651 ± 0.035^aA^	18.12 ± 1.08^a^
Dyslipidaemic control (DL)	0.844 ± 0.025^bA^	0.839 ± 0.018^bA^	0.849 ± 0.036^cA^	25.96 ± 1.42^c^
DL + Atorvastatin (10 mg/kg bw)	0.833 ± 0.039^bB^	0.734 ± 0.027^abA^	0.682 ± 0.029^aA^	20.45 ± 0.94^ab^
DL + Atorvastatin (10 mg/kg bw) + Garlic (1% w/w in feed)	0.792 ± 0.019^bA^	0.791 ± 0.032^bA^	0.823 ± 0.037^bcA^	22.39 ± 0.46 ^b^
DL + Atorvastatin (5 mg/kg bw) + Garlic (0.5% w/w in feed)	0.802 ± 0.010^bA^	0.797 ± 0.052^bA^	0.807 ± 0.033^bcA^	21.75 ± 0.98^b^
DL + Atorvastatin (7.5 mg/kg bw) + Garlic (0.25% w/w in feed)	0.807 ± 0.042^bA^	0.750 ± 0.034^abA^	0.729 ± 0.033^abA^	20.09 ± 0.50^ab^
DL + Atorvastatin (2.5 mg/kg bw) + Garlic (0.75% w/w in feed)	0.823 ± 0.029^bB^	0.724 ± 0.042^abAB^	0.656 ± 0.037^aA^	17.97 ± 0.38^a^

Values are mean ± standard error on mean of eight observations; Means with different alphabets as superscripts differ significantly (*P*<0.05); ANOVA with Duncan’s multiple comparison; Capital alphabets for horizontal comparison and small alphabets for vertical comparison

The TBARS concentration (n moles malondialdehyde (MDA)/mg tissue) in kidney in the basal diet control was 18.12 ± 1.08, which was significantly (*P*<0.05) increased in DL (25.96 ± 1.42). The TBARS concentration in the treatment Groups 3 (20.45 ± 0.94), 6 (20.09 ± 0.50), and 7 (17.97 ± 0.38) was comparable with normal control [Table 1]. The Groups 4 (22.39 ± 0.46) and 5 (21.75 ± 0.98) showed significant (*P*<0.05) decrease when compared with Group 2, but significantly (*P*<0.05) higher when compared with normal control Group. Elevated concentration of TBARS suggests increased lipid peroxidation. The renal damage as evident from the findings of the study could be attributed to the increased lipid peroxidation due to atorvastatin following inhibition of its metabolizing enzymes and P-gp by garlic. It was earlier reported that garlic inhibits CYP3A4 and P-gp[12] with a possible increase in the drug concentration in the body. These results can be further substantiated from the histological and electron microscopy findings on kidney as mentioned above. The overview of results pertaining to renal parameters revealed that garlic offered protection especially in Group 7, which was treated with minimal dose of atorvastatin along with 0.75% w/w garlic in the feed, whereas garlic could not offer better protection in other Groups treated with high dose of atorvastatin. This may be due to the fact that the beneficial effects of garlic were masked due to the elevated concentration of atorvastatin, possibly due to the inhibition of the metabolizing enzymes or P-gp. The antioxidant and nephroprotective properties of garlic have been reported earlier by Pedraza-Chaverri *et al*.[13] against experimentally induced renal toxicity and oxidative stress. Segoviano-Murillo *et al*.[14] have attributed the renoprotective actions of garlic to S-allyl cysteine.

In conclusion, the study revealed that high dose of atorvastatin induces injury to the kidney if used either alone or in combination with high concentration of garlic, while low dose of atorvastatin in combination with high concentration of garlic has minimal nephrotoxic potential.
